# The Comorbidity of Endometriosis and Systemic Lupus Erythematosus: A Systematic Review

**DOI:** 10.7759/cureus.42362

**Published:** 2023-07-24

**Authors:** Ranim K Hamouda, Hadia Arzoun, Isra Sahib, Lisbeth Escudero Mendez, Mirra Srinivasan, Shoukrie I Shoukrie, Ravneet K Dhanoa, Ramaneshwar Selvaraj, Jyothirmai Malla, Tharun Yadhav Selvamani, Anam Zahra, Sathish Venugopal, Lubna Mohammed

**Affiliations:** 1 Internal Medicine, California Institute of Behavioral Neurosciences and Psychology, Fairfield, USA; 2 Internal Medicine, St. Bernards Medical Center, Jonesboro, USA; 3 Pathology, California Institute of Behavioral Neurosciences and Psychology, Fairfield, USA; 4 Orthopedics and Traumatology, California Institute of Behavioral Neurosciences and Psychology, Fairfield, USA; 5 Internal Medicine/Family Medicine/General Surgery, California Institute of Behavioral Neurosciences and Psychology, Fairfield, USA; 6 Internal Medicine/Family Medicine, California Institute of Behavioral Neurosciences and Psychology, Fairfield, USA; 7 General Surgery, California Institute of Behavioral Neurosciences and Psychology, Fairfield, USA; 8 Surgery, California Institute of Behavioral Neurosciences and Psychology, Fairfield, USA; 9 Neurology, California Institute of Behavioral Neurosciences and Psychology, Fairfield, USA

**Keywords:** multisystem involvement, rheumatology & autoimmune diseases, auto immune disease, systemic lupus erythematosus, general gynecology

## Abstract

Autoimmune diseases manifest in genetically predisposed individuals exposed to certain triggers that aggravate immune dysfunction and result in an exacerbated immune response in the form of hyperactivity to both the humoral and cell-mediated response. The devastating reality apart from the severity of the disease is that multiple immune diseases could co-occur, increasing the patient's physical, psychological, and financial burden. Autoimmune diseases are utterly deranging. One of the dreadful autoimmune diseases is systemic lupus erythematosus (SLE). SLE is a rheumatological disease that affects multiple systems, and there are no predictors to know which system will be affected in the future. It could affect the mucocutaneous system. It could also present with hematological, rheumatological, neuronal, renal, pulmonary, and cardiac manifestations. SLE is prevalent in females, predominantly in the childbearing age group. The pharmacological therapy and bombarding pathophysiology of the disease lead to obstetrical and gynecological complications such as infertility, abortion, miscarriage, and stillbirth.

Over the past decade, the autoimmune disease comorbidity increased eminently. One of the common associations is rheumatological diseases (like rheumatoid arthritis, Sjogren syndrome, and SLE) with gynecological diseases (e.g., endometriosis and uterine fibroids). SLE and endometriosis have strong associations, and the prevalence of each condition is relatively high among the female population.

*Endometriosis* is a chronic disease triggered by inflammation, hormonal milieu, and other predisposing factors that lead to the fibrous tissue that lines the uterus (endometrial tissue) to be implanted at sites other than the uterus, commonly in the peritoneum and mesentery. The pathogenesis of this association remains unexplained. The approved theory is that their immune dysfunction is summarized by the elevated humoral and cell-mediated response, which leads to an attack to the epithelium, mesothelium, and Serosa and leads to fibrous tissue deposition in different sites other than the uterus. Statistical evaluations have shown a remarkable association between autoimmune diseases and both gynecological and nongynecological diseases.

## Introduction and background

Systemic lupus erythematosus (SLE) is a multisystem chronic autoimmune disease. SLE is a chronic inflammatory disease with the autoactivation of T-cells and B-cells with the production of autoantibodies and with the production of the inflammatory cytokine infiltrating multiple systems, primarily the kidney, resulting in nephritis with the deposition of the immune complex in the glomerular tissue. Skin, joints, brain, lungs, and blood vessels can be affected as well. Therefore, SLE can be classified as a multisystem inflammatory disease. This disease is characterized by its clinical variability [1]. Its presentation ranges from mild-moderate mucocutaneous symptoms to a systemic involvement, including profound, severe manifestations in the central nervous system (CNS). The etiology behind this disease is unknown. However, it has been established that genetic predisposition, environmental triggers, hormonal milieu, and socioeconomic status levels all interact to cause the disorder [2]. The disease is more common in women of African American ethnicity [3]. It is predominant in the younger age group. However, it can still be present at 50 years old or more, and this has been clinically presented in 20% of this age group [4]. The disease is characterized by occurring in bouts as it has a remitting and a relapsing period [5]. Diagnosis would be based on laboratory and clinical investigations, and the management would be system-directed and symptom-oriented. The guidelines state that sunscreen, a healthy diet, regular exercise, and the avoidance of the risk factors that aggravate the disease, like smoking with the administration of an immunosuppressive and anti-inflammatory drug, would help control the disease [6].

**Figure 1:**
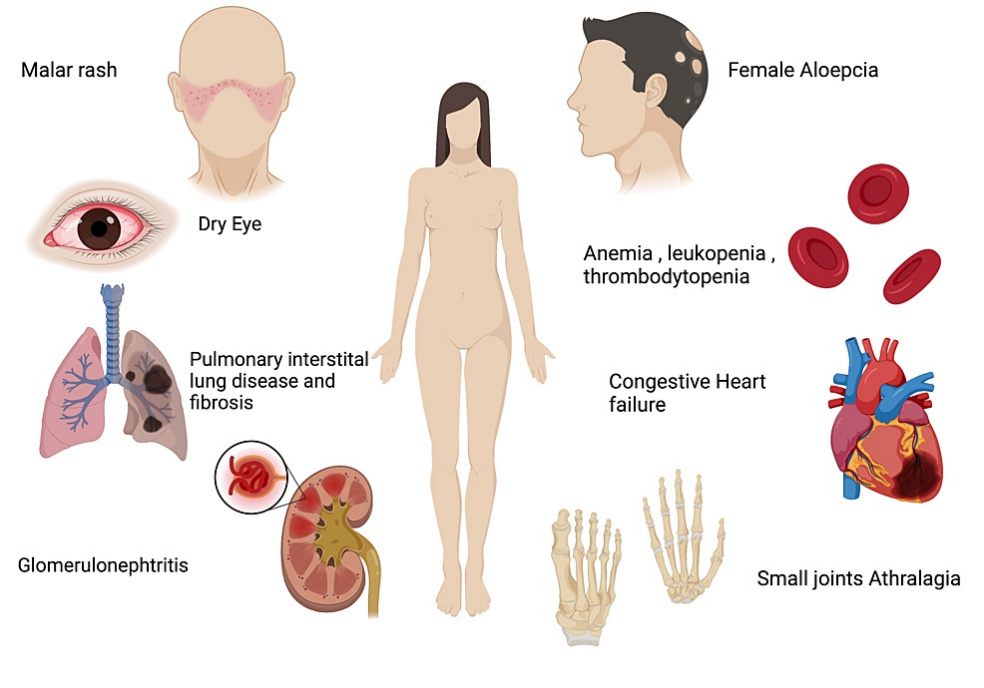
Clinical features in a systemic pattern that could potentially emerge in a patient with systemic lupus erythematosus. This figure was created with BioRender.com and approval was received for publishing the figure.

Endometriosis is a chronic inflammatory disease and is diagnosed based on the presence of endometrial tissue beyond the uterine lining [7]. Endometriosis would remain the most puzzling disease in gynecology as its etiology is unknown and affects about 5% to 10% of women [8]. It is the third most common gynecological cause of hospitalization [7]. It presents with severe pelvic pain, especially menarche, during menstruation and intercourse. Moreover, the patient would present with back pain and nausea. Infertility is a common symptom manifested with endometriosis [8]. A cross-sectional study concluded that 41% of the patients in the study were affected by infertility, and 99% had pelvic pain [9]. The diagnosis is confirmed only laparoscopically. The current treatment is analgesic for ovulatory pain. Surgery has successfully improved pregnancy rates and is the first choice for infertility treatment [8]. Even though the etiology of SLE and endometriosis is unknown, the incidence of endometriosis is high in patients with SLE. The lupus attacks become more intense and recurrent than those who did not present with endometriosis. The high incidence of autoimmune diseases and the interlink between the diseases should increase the physician's awareness about the possibility of other illnesses that might affect the quality of life [10]. Figure 1 illustrates the clinical features in a systemic pattern that could potentially emerge in a patient with SLE [8]. 

This study reviews the prevalence of endometriosis in patients with SLE. It compares the prevalence of endometriosis in patients with SLE to endometriosis in females without SLE. It presents the constitutional symptoms presented along with the current manifestations and future treatment regimens to improve the patient's quality of life. Current plans for the treatment of endometriosis associated with SLE include in vitro fertilization (IVF) implantation. They have shown successful results of a 30-week live birth and no complications recorded in the follow-up regarding lupus flare reaction, thrombosis, or ovaries hyperstimulation syndrome [11]. Furthermore, interventions were prescribed for patients with SLE, including hydroxychloroquine. It proved to be a safe drug to be administered during pregnancy to decrease the autoimmune reaction and the bouts of the lupus attack, thrombus caused by the antiphospholipid antibodies, in addition to the reduced need for steroid usage [12]. However, there is not any established method to prevent the development of endometriosis among patients with SLE.

Methodology

The method used for this systematic review followed the guidelines of Preferred Reporting Items for Systematic Reviews and Meta-Analyses (PRISMA) [13].

Inclusion and exclusion criteria

The criteria specified for this review included case-control, cohort, clinical trial, systematic review, and cross-sectional studies. The studies included were those published from 2011 to 2021. The papers that have been chosen are those written in the English language. The population group selected was female, after puberty up to postmenopausal (12-60 years). The population group should answer the research question through the patient/population, intervention, comparison, and outcomes (PICO) format. Papers that were published in any language other than English, outside the age group, and included confounding factors that may affect the result of the review were excluded.

Information sources and search strategy

The database used were PubMed, PubMed Central (PMC), Google Scholar, Web of Science, Cochrane, and Base. The studies included in this review were based on keywords that created the Medical Subject Heading (MeSH) strategy in PubMed and applied a set of keywords in other databases. The relevant articles were then finalized by filtering records according to the titles and abstracts, after which a thorough analysis was done on all the subheadings of the articles. 

Keywords

The keywords that were included in the search study were endometriosis or endometrioma or adenomyosis, autoimmune, genetics, diagnostic imaging, immunology, pathophysiology, systemic lupus erythematosus, chronic lupus erythematosus, cutaneous lupus erythematosus or discoid lupus erythematosus or primary immunodeficiency or autoantibodies, lupus erythematosus classification, drug therapy, diet therapy, prevention and control, rehabilitation. Moreover, these keywords were also incorporated into the MeSH strategy in PubMed.

Data extraction and selection process

The quality appraisal tools were used primarily to ensure the studies' validity and check if the selected articles satisfied the inclusion criteria. Two writers have worked individually to extract the data following the specified criteria. Certain disagreements arose during the process. Those disagreements were resolved simultaneously. The data extraction was based on the gender (female), age group (mainly pubertal, childbearing period, and postmenopausal), and the association with other rheumatological and autoimmune diseases.

Quality assessment

The following studies have undergone an assessment for quality. The following reports were selected according to the PRISMA tool (a systematic review) and Newcastle-Ottawa (case-control and cohort study). A total of nine studies were assessed. Table 1 summarizes the type of study and the quality appraisal tools used for each study analyzed in this review. 

**Table 1:** Type of study and the quality appraisal tools used for each study analyzed in this review. PRISMA, Preferred Reporting Items for Systematic Reviews and Meta-Analyses

Type of study	Tools used	Number of studies
Systematic review	PRISMA	2
Case-control	Newcastle-Ottawa	2
Cohort	Newcastle-Ottawa	5

Results

**Figure 2:**
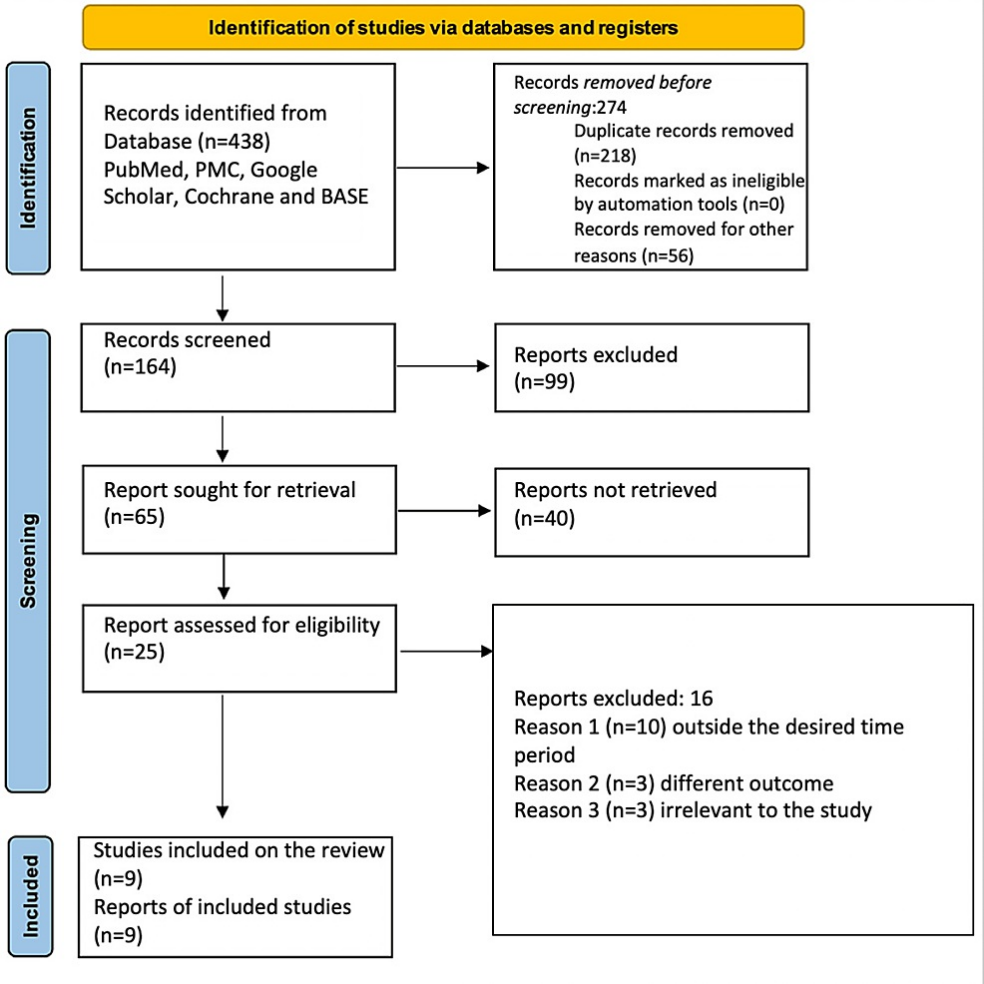
Depicts the search strategy used in this review in the form of a PRISMA flow diagram. PRISMA, Preferred Reporting Items for Systematic Reviews and Meta-Analyses

The search strategy was fulfilled through the following five databases: PubMed, PMC, Cochrane, Google Scholar, and Base. The limit set for selecting the articles was more than 70%. The initial search yielded 438 published articles, which were brought down to 164. This is due to the presence of 218 duplicate articles, and 56 were removed for other reasons. The remaining 164 records were further filtered to a total of 65 after screening and removed 99 records that did not match our research question or the stated inclusion or exclusion criteria. The remaining 65 articles were further condensed to 25 articles as 40 articles were not retrievable. The 25 articles came down to a final nine articles that were included in our systematic review. Out of the 16 articles that were removed, 10 were excluded because they were published outside the desired publishing period, three articles measured different outcomes, three articles were deemed irrelevant to the study, and nine articles were ultimately included for analysis. Figure 2 depicts the search strategy used in this review in the form of a PRISMA flow diagram [13]. 

Table 2 presents a summary of the results of all the studies included in this review.

**Table 2:** Summary of results for all the studies in this review.

Author	Year	Title	Method	Subject	Conclusion
Nielsen et al. [14]	2001	The Co-occurrence of Endometriosis with Multiple Sclerosis, Systemic Lupus Erythematosus and Sjogren Syndrome	Cohort Study	37,661	This study concluded that women with systemic lupus, multiple sclerosis, and Sjogren syndrome are more likely to develop endometriosis and other autoimmune diseases.
Muthuppalaniappan et al. [15]	2016	Silent Obstruction in a Young Woman with Systemic Lupus Erythematosus: A Case Report and Literature Review on Kidney Injury from Ureteral Endometriosis	Case Report	1	Patients with systemic lupus erythematosus and more likely to develop ureteral endometriosis.
Harris et al. [16]	2016	Endometriosis and the Risk of Systemic Lupus Erythematous and Rheumatoid Arthritis in the Nurses’ Health Study II	Cohort Study	16,578	There is a strong etiological correlation between endometriosis and the likelihood of developing systemic lupus erythematosus and rheumatoid arthritis.
Harris et al. [17]	2016	Endometriosis and Systemic Lupus Erythematous: A Population-Based-Case-Control Study	Case-Control Study	2,834 cases and 14,164 control cases	There is a significant correlation between SLE and endometriosis. Endometriosis could be a risk factor predisposing to systemic lupus erythematosus pathogenesis, or a certain etiology could be common between both diseases.
Matalliotaki et al. [18]	2018	Co-existence of Endometriosis with 13 Non-gynecological Co-morbidities: Mutation Analysis by Whole Exome Sequencing	Cohort Study	1,000	Gene sequencing is considered a vital predictor technique, showing a compelling association between endometriosis with systemic lupus erythematosus and other autoimmune diseases.
Shigesi et al. [11]	2019	The Association Between Endometriosis and Autoimmune Diseases: A Systematic Review and Meta-Analysis	Systematic Review and Meta-Analysis Study	N/A	Various study types have concluded a strong correlation between endometriosis and rheumatological autoimmune disease.
Farland and Harris [19]	2020	Long-Term Health Consequences of Endometriosis – Pathways and Mediation by Treatment	Systematic Review	NA	The knowledge behind the mechanism of association between endometriosis and other autoimmune diseases/ chronic diseases is limited.
Fan et al. [20]	2021	Association Between Endometriosis and Risk of Systemic Lupus Erythematosus	Retrospective Cohort Study	16,758 with endometriosis and 16,758 without endometriosis	Multiple etiological factors contribute to the comorbidity.
Cozier et al. [21]	2021	A Prospective Study of Reproductive Factors in Relation to Risk of Systemic Lupus Erythematosus Among Black Women	Cohort	125	The late menstrual age or breastfeeding for more than or equal six months would increase the likelihood of systemic lupus erythematosus.

## Review

Discussion

In this section, the etiology, pathophysiology of SLE, and endometriosis, along with a statistical evaluation, are discussed. A note on the treatment and prevention strategy and the limitations of this study are also enumerated. 

Etiology

The etiology of autoimmune diseases and their comorbidity remain unknown. The main etiological component is the hereditary and genetic factors. According to previous statistical surveillance, 66% of the cases of SLE patients had a positive family history. A specific study illustrated a pathway explaining the correlation between endometriosis development and how it influences other diseases to be encountered. Endometriosis is induced via an altered environment in terms of inflammatory, immunological, or hormonal changes, in addition to shared risk factors, which could be genetic, ethnic, or other environmental factors supporting these risk factors. Furthermore, the treatment prescribed to treat endometriosis could be the leading cause of chronic disease, including autoimmune diseases like SLE and rheumatoid arthritis. These treatment methods that trigger other conditions include oral contraceptive pills (OCPs), hysterectomy, and oophorectomy. These treatment methods initiate an inflammatory cascade, immune dysregulation, and hormonal dysfunction [19].

Primarily, the diagnosis of endometriosis will be confirmed based on an investigation of the discharge sample. However, it was observed that the women who tested positive for endometriosis and reported SLE later was the same magnitude as women who performed laparotomy for endometriosis diagnosis. Ironically, the age of both groups was similar. However, the strongest association was among women who completed a previous hysterectomy. It was found that symptoms would be more profoundly severe than those who did not perform the procedure [17]. Identification of endometriosis along with connective tissue diseases like SLE and rheumatoid arthritis will be demonstrated in a two-stage procedure in a case-control study.

To begin with, women who tested positive for endometriosis filled out a screening questionnaire. Women at risk for testing positive for connective tissue diseases filled out a screening questionnaire every other year guided by the Connective Tissue Disease Screening Questionnaire (CSQ). Women who screened positive will be asked to consent for their medical file to be reviewed by a rheumatologist, preferably two rheumatologists. This incident case report was done by two board-certified rheumatologists who followed the American College of Rheumatology (ACR) diagnostic criteria for SLE. The cases were confirmed by both doctors via the ACR criteria. The guaranteed case rate for comorbidity of endometriosis along with SLE was 69%. At the time of the report, there was 7% of the cases diagnosed positive for SLE. The screening results were similar in another prospective cohort study (The Iowa Women Health Study). Those two studies highlighted the risk of CSQ disease occurrence at the time diagnosed with endometriosis. Incident reports were not exclusively positive for the patients at the time of the depiction of CSQ [16].

Specific confounders were found to be profoundly associated with endometriosis and autoimmune diseases comorbidity. Those confounders are classified into nonmodifiable factors (risk traits) and modifiable factors (social characteristics). The risk factors include menarche age, the average duration of the menstrual cycle, the number of pregnancies, the ratio of parity to total breastfeeding time, infertility, hysterectomy, oophorectomy, and last but not least, race and ethnicity. The social factors include body mass index, physical activity, smoking, usage of OCPs, post-menopausal hormones, and routine excessive consumption of analgesics [16].

Statistical evaluation

The US Endometriosis Association Female Membership proposed the results from a survey that stated that women diagnosed with endometriosis had a higher risk than other women in terms of developing SLE and other autoimmune diseases, and this was supported by the prevalence odds ratio for SLE over 20 cases in comparison with a female control group from the general population in a case-control study [17]. A systematic review reported that the prevalence was relatively high, especially in women under 45 years old, with a hazard ratio equivalent to 2.11 and a confidence interval between 1.06 and 4.19. Also, the history of infertility poses a hazard ratio of 2.41 and a confidence interval of 2.11 to 5.24. However, those factors seem statistically insignificant as the *P*-values were 0.86 and 0.44 for both factors, respectively [16].

In a prospective study, 954,476 individuals and 125 cases were diagnosed as SLE after follow-up. Those cases shared late menarche and prolonged breastfeeding period. Regarding the age of menarche, it was statistically evaluated to show a hazard risk of 2.31 and a confidence interval lying within 1.30 to 4.11 at or more than 15 years in comparison with 12 years. Moreover, the hazard risk for the breastfeeding duration was 1.73, and the confidence interval was between 1.01 and 2.94 if the course was more than six months. This study denied any relation between the number of parities, age of first parity, hysterectomy, or the current menstruation status with comorbidity of SLE and endometriosis [21].

A cross-sectional study was performed on women diagnosed with endometriosis from the nation's endometriosis support organization compared with controls from the US general population. The study concluded that the prevalence odds ratio for SLE was 20.7; the confidence interval was between 14.3 and 29.9 [16]. The most recent study was a retrospective cohort study in Denmark, which stated that the risk of SLE in women diagnosed with endometriosis is significantly higher than in the general population. The incidence ratio was 1.6, with a confidence interval between 1.2 and 2.1, and this study also confirmed the fact that risk could be attenuated when endometriosis is treated properly via the modified surgical procedure [16].

A systematic review published in 2019 reported that a cross-sectional study showed that women diagnosed with SLE are at a higher risk for endometriosis than the general female population. The prevalence was 20.7, with a confidence interval of 14.3 to 29.9, and it was clinically significant at the *P*-value <0.01. The high prevalence rate reported was mainly due to the self-consciousness of the patients, as most of the patients were recruited from the Endometriosis Patient Association [18]. A case-control study was performed to confirm that the association between endometriosis and SLE is different from the prevalence rate between uterine fibroids and SLE. The prevalence was significantly higher; the 9% prevalence rate of SLE in endometriosis-positive females versus the 0% prevalence rate in the case of having uterine fibroids. However, this study could have been biased as the study group was pretty small [18]. In 2011, a study analyzed that 9,191 females were diagnosed with endometriosis via the gold standard laparoscopy/laparotomy procedure. It stated that the association with autoimmune diseases like multiple sclerosis (MS) is significantly higher than with SLE [14].

Certain diseases increase the risk of endometriosis. Those diseases could be hypertension, dyslipidemia, hepatic diseases, cardiovascular diseases, pulmonary disease, and certain medication intake, which include hormones, nonsteroidal anti-inflammatory drugs (NSAIDs), and corticosteroid usage. According to this study, those factors increase the association between endometriosis and chronic diseases, and this was a statistically significant association as the *P*-value <0.05 [20].

Pathophysiology

Women diagnosed with endometriosis have shown a decline in natural killer cell cytotoxicity and elevation in both the number and activation of macrophages. Endometriosis-positive women have shown diminished cell-mediated response and elevated humoral response compared to women without endometriosis. Those immune disturbances play a role in the initiation of diverse autoimmune diseases. These are multiple theories explaining the pathophysiology of endometriosis. The two accepted theories include Sampson's theory of retrograde menstruation, which states the endometrial tissue would be implanted on the extrauterine tissue, and the plaque theory that says the ovarian epithelium in particular, or the mesothelium of the pelvic peritoneum. The primary key to the pathophysiology's hypotheses is inflammation; the coelomic metaplasia pathway is still unexplained. After endometriosis has occurred, a cascade of inflammation would be initiated, leading to adhesions and scar formation on top of the peritoneal tissue surfaces. This would result in the elevated release of prostaglandins, cytokines, and other inflammatory products like metalloproteinases, chemokines, etc. These products would result in the deposition of fibrin and further scarring and adhesions. The excessive inflammation imbalance and immune instability enhance the extra-endometrial tissue to survive and the development of other immune conditions, including SLE, MS, Sjogren syndrome, and rheumatoid arthritis [19].

Furthermore, other studies have correlated endometriosis with other autoimmune conditions with elevated synergic circulating hormones, mainly steroids. Case reports have proposed the hypothesis of the development of endometriosis with SLE that further proves the theory of the imbalance of humoral and cell-mediated immune response. Immune surveillance was performed on women with endometriosis that concluded that elevated immunoglobulins (Ig) like IgG, IgM, IgA autoantibodies, and anti-endometrial auto-antibodies depressed cell-mediated immunity in terms of cellular activity. However, according to this study's result, the number of T-cells, B-cells, and natural killer cells was elevated [16].

**Figure 3:**
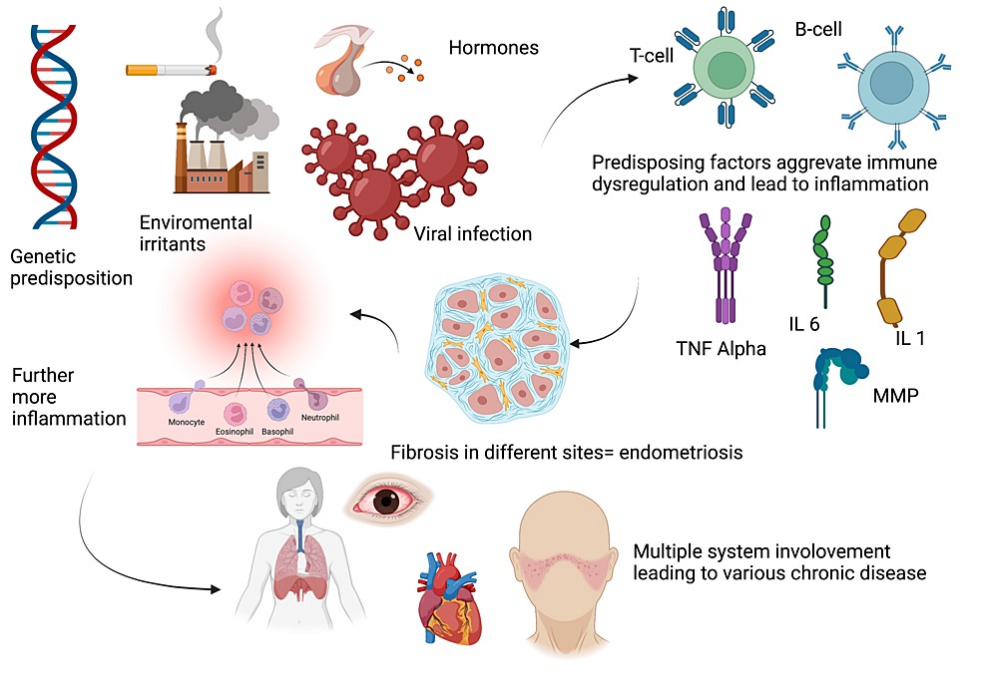
Pathophysiology of endometriosis and other nongynecological chronic autoimmune disease. This figure was created with BioRender.com and approval was received for publishing the figure. MMP, matrix metalloproteinase; TNF, tumor necrosis factor; IL, interleukin

Moreover, a specific antibody was observed in comorbidity of SLE and endometriosis, known as the anti-nuclear auto-antibody. The immune surveillance found that particular cytokines are dominant in this case. The dominant inflammatory cytokines are interleukin 1, interleukin 6, and tumor necrosis factor. This immune response heightens the chance of developing the extrauterine endometrial tissue, further aggravating the immunological disease, mainly SLE [16]. The association of endometriosis with nongynecological, autoimmune diseases like SLE was found in individuals with susceptible gene loci confirmed via several case-control studies and by the genome-wide association studies (GWAS) [18]. Figure 3 demonstrates the pathophysiology of endometriosis and other nongynecological chronic autoimmune disease

A prospective follow-up with the future generation's gene sequence allows a broad perspective on the primary gene susceptibility loci that increase the risk of comorbidity incidence. This study was performed on three generations diagnosed with endometriosis via a surgical confirmation and then consented to a whole-genome sequence and found that they were at risk for 13 diseases; those diseases were mainly nongynecological [18].

The gene sequencing detected that risk groups are homozygous for the T allele for SLE on the protein tyrosine phosphatase, non-receptor type 22 (PTPN22) gene, which codes for the tyrosine phosphatase protein. This protein stimulates the PTPN22 gene, which leads to the simulation and modification of the T cell's adverse selection on the thymus and inhibition of the auto-active T cells in the systemic circulation. The association is yet not clear. However, females diagnosed with endometriosis had common gene sequencing risk loci. Positive autoimmune individuals presented with a heterozygous gene were more likely to show SLE as a nongynecological autoimmune disease [18].

Prevention and treatment strategies

It was noted that the association could be attenuated if there was an appropriate modification in the hysterectomy/oophorectomy procedures [16]. Hysterectomy is the first line for treating severe endometriosis, and in certain conditions, endometriosis is diagnosed during the procedure. Hence, the risk of comorbidity would remain present but could only be attenuated in the situation where the procedure modifications were followed [17]. Furthermore, women diagnosed with endometriosis would most probably have a hysterectomy earlier than other women. This will result in surgical menopause, and the early hormonal imbalance would lead to autoimmune disease induction or aggravation to an excessive disease [16]. Moreover, endometriosis's first-line treatment is OCPs, which pose an immediate risk for SLE incidence. Theoretically, the cessation of OCP usage would alter the association. However, clinically, there were no significant observed changes in drug cessation [16].

A systematic review stated that the central generally accepted hypothesis is the theory of retrograde menstruation. This theory proposes that the endometrial cells will be disseminated and expressed through the uterine tubes. This phenomenon occurs in a significant proportion of females. However, few individuals will be diagnosed with endometriosis except those with identifiable risk factors, immune dysregulation, and imbalanced hormonal milieu. Immune dysregulation and imbalanced hormonal milieu result in excessive endometrial tissue deposition into ectopic sites. Further dysregulation in the immune system leads to the development of autoimmune disease (almost 13 nongynecological autoimmune diseases could develop) [18].

Furthermore, multiple pathways initiate autoimmune disease as these endometrial cells escape the immune surveillance. Endometrial cells will be widely spread beyond the uterine wall and into the peritoneum. The immune cells analysis showed that the neutrophils and macrophages of the peritoneum are widely elevated with a declined function of the cytotoxic cells, killer cells, and elevated autoantibodies. Certain blood and urinary biomarkers were found to be specific for endometriosis, mainly the endometrial autoantibodies and interleukin 6. Moreover, proving the hereditary involvement was the gene sequencing that was performed. The risk gene was PTPN22, which showed a significant association with the disease comorbidity [18].

Limitations

There are profound limitations to this research question. To begin with, the explanation of the disease etiology and pathophysiology is based mainly upon hypothesis. The fact that SLE is a vast disease regarding its manifestation makes it harder to investigate significantly. The definitions of the disease itself may differ regarding a previous laparoscopic procedure or hysterectomy. Furthermore, there is a lack of prestigious and trustworthy studies that have extensively addressed this issue. In addition, there is a significant degree of bias and inadequate follow-up with the cases. Moreover, certain published articles have exhibited poor quality in terms of the quality assessment analysis. This can be attributed to the small sample size and the inability of researchers to determine the chronological occurrence of simultaneous diseases, as well as the similarity or dissimilarity in disease etiology or pathophysiology between the conditions.

## Conclusions

Autoimmune diseases are very diverse and can occur spontaneously without known pathogenesis or specific etiology. Autoimmune diseases occur in individuals with inherited, genetic predispositions, which cause immunological dysfunction. Different factors initiate and aggregate the illness and increase the association. SLE occurs in females aged 40 years. However, clinically, SLE is observed throughout the childbearing period. Endometriosis occurs as well in the same age group. Both diseases result in significant difficulty regarding pregnancy as it is very likely for abortion, miscarriage, and stillbirth.

SLE and endometriosis are very prevalent in the female population, and there is excellent comorbidity between both diseases. Endometriosis is diagnosed laparoscopically and is highly common in females who have undergone hysterectomy. However, adequately modified hysterectomy decreases postoperative inflammation, immune irritation, and fibrosis, leading to autoimmune diseases in susceptible individuals. Specific therapies are being proposed to limit the disease, which can be modified hysterectomy as a surgical therapy and anti-inflammatory like corticosteroids and NSAIDs to limit the disease progression.
